# Social-cognitive determinants of the tick check: a cross-sectional study on self-protective behavior in combatting Lyme disease

**DOI:** 10.1186/s12889-017-4908-1

**Published:** 2017-11-25

**Authors:** Amy van der Heijden, Bob C. Mulder, P. Marijn Poortvliet, Arnold J. H. van Vliet

**Affiliations:** 1 Strategic Communication Group, Wageningen University, Hollandseweg 1, 6706 KN Wageningen, The Netherlands; 2 Environmental Systems Analysis Group, Wageningen University, Droevendaalsesteeg 3, 6708 PB Wageningen, The Netherlands; 3 Strategic Communication Group, Wageningen University, P.O. Box 8130, 6708 PB Wageningen, The Netherlands

**Keywords:** Lyme disease, Tick check, Determinants, Health behavior, Social cognitive theory

## Abstract

**Background:**

Performing a tick check after visiting nature is considered the most important preventive measure to avoid contracting Lyme disease. Checking the body for ticks after visiting nature is the only measure that can fully guarantee whether one has been bitten by a tick and provides the opportunity to remove the tick as soon as possible, thereby greatly reducing the chance of contracting Lyme disease. However, compliance to performing the tick check is low. In addition, most previous studies on determinants of preventive measures to avoid Lyme disease lack a clear definition and/or operationalization of the term “preventive measures”. Those that do distinguish multiple behaviors including the tick check, fail to describe the systematic steps that should be followed in order to perform the tick check effectively. Hence, the purpose of this study was to identify determinants of systematically performing the tick check, based on social cognitive theory.

**Methods:**

A cross-sectional self-administered survey questionnaire was filled out online by 508 respondents (M_age_ = 51.7, SD = 16.0; 50.2% men; 86.4% daily or weekly nature visitors). Bivariate correlations and multivariate regression analyses were conducted to identify associations between socio-cognitive determinants (i.e. concepts related to humans’ intrinsic and extrinsic motivation to perform certain behavior), and the tick check, and between socio-cognitive determinants and proximal goal to do the tick check.

**Results:**

The full regression model explained 28% of the variance in doing the tick check. Results showed that performing the tick check was associated with proximal goal (β = .23, *p* < 0.01), self-efficacy (β = .22, *p* < 0.01), self-evaluative outcome expectations (β = .21, *p* < 0.01), descriptive norm (β = .16, *p* < 0.01), and experience (β = .13, *p* < 0.01).

**Conclusions:**

Our study is among the first to examine the determinants of systematic performance of the tick check, using an extended version of social cognitive theory to identify determinants. Based on the results, a number of practical recommendations can be made to promote the performance of the tick check.

**Electronic supplementary material:**

The online version of this article (10.1186/s12889-017-4908-1) contains supplementary material, which is available to authorized users.

## Background

Lyme disease, also known as Lyme borreliosis, is the most prevalent tick-borne disease in Europe. It is primarily transmitted through a bite from a tick that is infected with the *Borrelia burgdorferi* bacteria [1]. When left untreated, Lyme disease may cause severe and potentially chronic symptoms such as neurological diseases, joint problems, and heart diseases [2].

Both societal and scientific issues call for further research to build effective public health programs to prevent Lyme disease. The first societal issue is the sharp increase in the incidence of tick bites and tick-borne diseases [3, 4]. As an example from the Netherlands, in 1994, 191 per 100,000 Dutch inhabitants visited their GP concerning a tick bite; a number that had risen to 488 per 100,000 inhabitants in 2014, although this now seems to stabilize [5]. Second, various studies show that many people do not take measures to prevent getting bitten by ticks and thus prevent Lyme disease [6–8].

Basic measures to prevent a tick bite include wearing clothing that minimizes skin exposure, for example by wearing long trousers, preferably tucked into socks [8], tucking in shirts and wearing closed shoes [9], using insect repellent on the body, and avoid walking in high grass and bushes [8, 9].

A particularly effective preventive measure, is to check the body for ticks and tick bites (henceforth called doing a tick check) after visiting areas where ticks could be present [8, 10]. The tick check is the only measure that can fully guarantee whether one has been bitten by a tick and provides the opportunity to remove the tick as soon as possible, thereby greatly reducing the chance to contract Lyme disease. The Dutch ‘National Institute for Public Health and the Environment’ explains in a video how to do a tick check systematically [11]. The first step is to remove one’s clothes for an overall visual inspection. The second step would be to systematically scan the body, for example from top to bottom, and paying extra attention to the warm places of the body, such as armpits, knees, under the underwear, and around the hairline in the neck. For the back or for other places one is not able to look at well, a mirror could be used. If a tick is found, it should be removed with pointy tweezers or a tick remover. One needs to grab the head of the tick with the tweezers and pull it out straight. If a tick remover is used, the instructions that come with that remover should be followed.

A systematic perusal of the literature^1^ reveals three issues in this field of study that may hamper the development of effective interventions. First, the effectiveness of interventions that aim to prevent tick bites is generally unknown [8]. Second, studies on behavioral determinants often lack a clear definition of the term “taking preventive measures” (e.g. [6, 12, 13]). This is problematic, because different behaviors have different causes (i.e. determinants), and effective behavior change interventions need to be based on a specific analysis of behavior determinants [14]. Moreover, different interpretations of the behavior could exist among researchers and research participants, further complicating the assessment of behavioral causes and consequents. Finally, the use of behavioral theories in current interventions is very limited [8]. This is problematic, because theories provide a basis for understanding behavior, which allows for systematic development and evaluation of interventions, as well as accumulation of evidence of what is effective and what is not [14, 15].

As the tick check was identified as a key recommended behavior to prevent tick bites and thus Lyme disease, the aim of the present research is to improve the understanding of the determinants for performing the tick check. Using guidelines for systematic intervention development [14], social cognitive theory (SCT) [16, 17] is chosen as the explanatory theory for a number of reasons (for a schematic overview of SCT, see [18], p.146). Self-efficacy is the pivotal determinant in SCT, which is the belief a person has about his or her ability to perform the behavior, in this case the tick check. More specifically, self-efficacy means feeling capable of performing all the steps of the tick check. SCT predicts that only if the person feels capable of doing all this *correctly* and *consistently* after having visited nature (i.e. the person has high self-efficacy), this person sets the goal to do the tick check, as well as follows up on this goal. Self-efficacy is likely to play a central role, because the tick check consists of multiple, consecutive steps, which together require some skills, motivation, as well as environmental conditions (e.g. privacy, certain tools) that have to be organized by the actor. Several studies show that self-efficacy affects taking preventive measures to avoid tick bites and Lyme disease [2, 19–21]. Mowbray et al. ([20], p.398), for example, found that high self-efficacy towards removing a tick was associated with checking for ticks after walking (OR 2.13; 95% CI 1.64–2.75).

SCT also specifies how self-efficacy influences three other determinants that have unique and additional effects on behavior, which are outcome expectations (physical, social and self-evaluative), sociostructural factors (facilitators and impediments), and setting proximal goals. Although using different labels for these determinants, various studies provide evidence for their effects on preventive behaviors.

For example, outcome expectations are examined in nine studies [2, 6, 12, 13, 19–23]. Herrington et al. [6] describe this determinant as attitudes that favor personal protection against Lyme disease, whereas Shadick et al. [21] mention the belief that preventive measures’ benefits are more important than the inconvenience that they cause. These are both examples of physical outcome expectations, and, more specifically response efficacy, because these beliefs concern the efficacy of the response, i.e. the extent to which preventive measures are believed to be effective.

Notably, the majority of studies focus on two types of beliefs that can be interpreted as two other, specific forms of physical outcome expectations [6, 12, 13, 20–23]. These are perceived severity and perceived susceptibility, theoretical concepts from the health belief model [24], which together constitute perceived threat. Theoretically, perceived threat leads to health-promoting behavior, such as the tick check. Hallman et al. [12] found, for example, that the belief that Lyme disease is not easy to cure (i.e. severe) is positively associated with taking preventive measures (*r* = .16, *p* < .02). Herrington [13] also included the belief ‘being concerned about being bitten by a tick’ and found that people who were very concerned (i.e., high perceived susceptibility) are more likely to have taken precautions against tick bites than people who are less concerned (OR 11.2; 95% CI 5.8–21.8 and OR 4.4; 95% CI 1.3–15.3; p.138). Thus, together with response efficacy, perceived susceptibility and severity represent relevant specifications of physical outcome expectations, as has also been noted by Bandura [17].

This can be related to five studies [2, 10, 12, 13, 22] in which people’s own experiences with ticks and Lyme disease were included as a determinant, because these factors may raise both perceived severity and susceptibility, as well as the response efficacy. For example, Beaujean et al. ([2], p.232) found that having experienced tick bites in the past was a significant predictor of checking the skin for ticks (OR 2.19; 95% CI 1.27–3.78).

Knowledge is theoretically related to outcome expectations, because beliefs are often based on what a person knows about that behavior, such that “knowledge creates the precondition for change” ([17], p. 624). For example, knowledge about Lyme disease and the tick check has to be acquired before outcome expectations can be formed. However, knowledge is typically only indirectly, and thus weakly related, to behavior, leading to more mixed evidence for its effects. For example, whereas Herrington et al. [6] found that higher levels of knowledge about Lyme disease were associated with taking preventive measures (OR 1.5; 95% CI 1.2–1.9), Shadick et al. [21] did not. That is because people *evaluate* what that knowledge means in terms of costs and benefits of the behavior (i.e. outcome expectations) associated with that knowledge [17].

Finally, impediments to taking preventive measures have also been studied. Mowbray et al. [20] conclude that having little time and forgetting present barriers to taking measures to prevent Lyme disease.

In sum, our review of the literature shows evidence for SCT constructs, as well as noteworthy additions. The emphasis has been on examining the behavioral effects of knowledge about ticks and Lyme disease, and related physical outcome expectations, often studied under the headers of perceived susceptibility and severity. Thus, the aim of the present study is to provide a comprehensive understanding of the factors that underlie systematic performance of the tick check, using SCT as the explanatory framework. Perceived susceptibility and severity will be incorporated as context-specific categories of physical outcome expectations, whereas knowledge and previous experience with ticks and Lyme are additional determinants.

## Methods

### Design and procedure

The research was set-up as a cross-sectional study, using a self-administered survey questionnaire. The questionnaire was distributed through the internet using Qualtrics survey software. The survey invitation was spread through Facebook, as well as via the websites Naturetoday.com and Tekenradar.nl, which attract approximately 60,000 and 70,000 unique visitors each month respectively during the summer season. Nature Today presents news items about nature, thus the target group is people who are interested in nature. Tekenradar.nl is a citizen science website that presents information about ticks, Lyme disease and preventive measures, and allows people to report tick bites and Lyme disease cases. Our aim was to recruit people who visit nature, because they represent a target group that is more likely to get bitten by ticks, and thus these people should perform the tick check after their visit.

### Participants

A power calculation indicated that at least 194 participants were required (alpha = .05; power of (1 – β) = 80%; expected correlation *r* = 0.2). In total, 508 participants were included in the analysis through convenience sampling. Criteria for inclusion were being an adult (18 years or older) and completing the entire questionnaire. It was not possible to calculate the response rate, because the total amount of exposures to our survey invitation was unknown.

Regular visitors of nature may have been overrepresented in this sample, due to the content of the websites used for recruitment. However, these people therefore represent a group who is more at risk of contracting tick bites and Lyme disease, and thus in need of preventive action.

### Measures

Self-reported data was collected. The questionnaire contained scales for the behavior (i.e. doing the tick check) and for each determinant (i.e. self-efficacy, outcome expectations, knowledge, experience, perceived susceptibility and severity, and proximal goals). Every determinant was measured using a specific scale, each consisting of multiple items. Existing scales with known validity were used whenever available, translated into Dutch by the authors, and revised in order to measure beliefs about ticks, Lyme disease and doing the tick check. Based on Bandura’s [25] recommendations for measuring SCT determinants, a hundred point scale was used for all psychological scales. The English questionnaire is included as an Additional file 1.

Four items were used to measure the current performance of the tick check, on a 100-point scale from ‘never’ to ‘always’. Scale reliability was determined through calculating Cronbach’s alpha, and reached high reliability (α = .84). An example of an item is ‘During the past month, when I had visited nature, I checked my body for ticks’.

Self-efficacy was measured with fifteen items (α = .91), on a 100-point scale from ‘certainly not’ to ‘certainly yes’. The design of this scale was based on a guide for the construction of self-efficacy scales [25]. Measures of impediments were included, because Bandura [17] states that efficacy beliefs should be measured against gradations of impediments to successful performance. Examples of items to measure self-efficacy are ‘I am able to perform the tick check when I am in company of other people’ and ‘I can perform the tick check completely if I have little time’. In addition to impediments being integrated in the self-efficacy items, there was an open question in the survey about impediments in which participants could fill in personal barriers to perform a tick check.

Knowledge regarding ticks, the tick check and Lyme disease was measured with ten statements that participants could answer with ‘true’, ‘not true’ or ‘I don’t know’, cf. [26]. For each correct answer the participants received one point, while a wrong answer and ‘I don’t know’ yielded zero points. This way, each participant received a knowledge score ranging from zero to ten. A knowledge item looked for example like ‘Ticks can be active at temperatures below fifteen degrees Celsius’ (which is true). In addition, there were three items to measure experience and the participants received one point for each experience that was true for them. A statement to measure experience was, for example, ‘I have been bitten by a tick before.’

Outcome expectations were measured with 18 items, on a 100-point scale from ‘totally disagree’ to ‘totally agree’. In line with SCT, three types of outcome expectations were measured, i.e. physical, social and self-evaluative.

Fourteen items were designed to measure physical outcome expectations, and among these, there were four items for perceived severity (α = .81), for example, ‘I think Lyme disease is a serious condition‘[27]. Four items measured perceived susceptibility; however, Cronbach’s alpha indicated low internal consistency (α = .49), which was deemed insufficient. Thus, even though items were based on existing scales [27], we were not able to reliably measure perceived susceptibility, which therefore was excluded from the analyses. Six items measured the response efficacy of doing the tick check, and were based on the SCT-questionnaire by Dewar et al. [28]; for example, ‘Doing a tick check prevents me from getting Lyme disease’. However, one negatively formulated item was deleted, because this increased Cronbach’s alpha from α = .69 to .76.

Social outcome expectations were measured with two items. Based on Cialdini, Reno and Kallgren [29], one item measured the injunctive norm (‘People whose opinion I value, will appreciate it if I do a tick check‘), whereas the second item measured the descriptive norm (‘People whose opinion I value, perform the tick check after having visited nature’). It is suggested that descriptive norms and injunctive norms differ with regard to their impact on behavior [30]. People could have a high score for injunctive norm and a low score for descriptive norm or the other way around. Therefore, both items were not merged into a single scale.

Finally, two items measured self-evaluative outcome expectations (α = .69); for example ‘By doing a tick check I feel good about myself’.

There were nine items to measure participants’ proximal goal to do a tick check (α = .89). The formulations were based on Ajzen’s [31] recommendations, using three gradations. For example, three gradations were ‘In the coming month, if I visited nature, I will try to do a tick check’; ‘In the coming month, if I visited nature, I really plan to do a tick check’; and ‘In the coming month, if I visited nature, I will definitely do a tick check’. In the last section of the questionnaire, demographic variables were measured such as age, gender and level of education.

### Statistical analyses

Mean item scores were calculated for all scales, except for knowledge and experience, for which the sum of scores was calculated. For each scale, inter item reliability was determined by calculating Cronbach’s alpha.

Bivariate correlations were computed to explore whether the determinants and behavior were associated as expected. Next, multivariate associations were tested by means of two separate hierarchical regression analyses. In Model 1, performing the tick check was the dependent variable (DV), and in Model 2 this was proximal goal.

The main categories of determinants (i.e. independent variables; IV’s) were entered as separate blocks. For model 1, proximal goal was entered in block 1; self-efficacy was added in block 2; all categories of outcome expectations in block 3; and knowledge and experience in block 4. For model 2, self-efficacy was added in block 1; in block 2, all categories of outcome expectations were added; and knowledge and experience were added in block 3.

Before running the final models, associations with potential confounders were examined for each model. Significant confounders (*p* < .05) were maintained in the analysis as a covariate.

## Results

### Respondent characteristics

In total, 964 online surveys were opened of which 512 were completed. One respondent was not adult (i.e. 17 years), and, together with three others who had missing values for age, was excluded from further analysis. A total of 508 participants were thus included in the analysis. Of that total, 255 were male (50.2%). Mean age of the sample was 51.7 years (SD = 16.0), ranging from 18 to 87 years.

Most participants (72.2%) had a higher education degree (higher vocational education or university), and 18.1% of the participants had done intermediate vocational school. For 9.4% of the participants, the highest educational level was high school and for 0.2% this was primary school.

The participants had different frequencies of visiting nature (forest, moorland, natural park or garden), but most participants visited nature daily or weekly (86.4%). 11.0% of the participants visited nature monthly, 2.2% visited nature a few times a year and 0.2% visited nature once or less than once a year.

The survey was distributed online through different websites. Most participants (67.9%) found the survey on the website Nature Today, followed by 18.7% who found it on Facebook, and 2.0% found the survey on Tekenradar. 12.3% filled in “Other” as source of where they found the survey.

### Correlations

Pearson correlations show that all determinants were associated in the expected directions with performing the tick check, except for perceived severity and injunctive norm (Table 1). The strongest correlates of doing the tick check were proximal goal and self-evaluative outcome expectations (both *r*’s = .34, *p* < .01). The weakest, significant correlates were experience (*r* = .19, *p* < .01) and knowledge (*r* = .14, *p* < .01).

**Table 1:** Pearson correlations between variables (*N* = 508)

	2	3	4	5	6	7	8	9	10
1. Performing tick check	.34^**^	.31^**^	.23^**^	.04	.08	.30^**^	.34^**^	.14^**^	.19^**^
2. Proximal goal	–	.11^*^	.34^**^	.13^**^	.23^**^	.22^**^	.49^**^	.00	.00
3. Self-efficacy		–	.29^**^	−.08	.12^**^	.24^**^	.18^**^	.17^**^	.16^**^
4. Response efficacy			–	.29^**^	.29^**^	.33^**^	.63^**^	.14^**^	.09^*^
5. Perceived severity				–	.20^**^	.08	.23^**^	.00	−.05
6. Injunctive norm					–	.31^**^	.39^**^	.02	.00
7. Descriptive norm						–	.40^**^	.05	.04
8. Self-evaluative							–	.01	.04
9. Knowledge								–	.45^**^
10. Experience									–

^**^ *p* < 0.01, ^*^ *p* < 0.05

### Multivariate associations

In model 1, with performing the tick check as DV, educational level was the only confounder that was significantly associated with the tick check, whereas gender, age, and nature visiting frequency were not (data not shown). As a result, only level of education was maintained as covariate throughout this regression analysis.

In the full model, explaining 28% of the variance in the tick check, proximal goal showed the highest beta coefficient (β = .23, *p* < .01), followed closely by self-efficacy (β = .22, *p* < .01), and self-evaluative outcome expectations (β = .21, *p* < .01) (Table 2). In contrast to expectations, none of the physical outcome expectations were significantly associated with tick check performance. Both social outcome expectations were significantly associated with the tick check, with descriptive norm being positively associated (β = .16, *p* < .01), and injunctive norm negatively (β = −.11, *p* < .01). Finally, experience (β = .13, *p* < .01), but not knowledge, was associated with performing the tick check.

**Table 2:** Regression analysis to test associations between SCT-determinants and performing the tick check (DV)

	β	*t*	*R* ^*2*^	*R* ^*2*^ change
DV: Performing the tick check				
Model 1:			.13	.11
Proximal goal	.33	7.99^**^		
Model 2:			.20	.07
Proximal goal	.30	7.57^**^		
Self-efficacy	.27	6.79^**^		
Model 3:			.26	.05
Proximal goal	.22	5.04^**^		
Self-efficacy	.24	5.89^**^		
Outcome expectations:				
Perceived severity	.02	0.40		
Response efficacy	−.08	−1.46		
Self-evaluative	.20	3.57^**^		
Injunctive norm	−.12	−2.68^**^		
Descriptive norm	.17	3.77^**^		
Model 4:			.28	.02
Proximal goal	.23	5.22^**^		
Self-efficacy	.22	5.31^**^		
Outcome expectations:				
Perceived severity	.02	.58		
Response efficacy	−.10	−1.87		
Self-evaluative	.21	3.74^**^		
Injunctive norm	−.11	−2.65^**^		
Descriptive norm	.16	3.81^**^		
Knowledge	.05	1.11		
Experience	.13	3.04^**^		

All models controlled for level of education (*R* ^*2*^ = 0.02); ^**^ *p* < 0.01, ^*^ *p* < 0.05

For model 2 (proximal goal is DV), only gender was the significant confounder, and was therefore maintained as a covariate throughout this regression analysis.

The full model explained 25% of the variance in proximal goal. Rather strikingly, self-evaluative outcome expectations (*β* = .43, *p* < .01) was the only variable showing a significant relationship with the proximal goal to perform the tick check in all three blocks (Table 3).

**Table 3:** Regression analysis to test associations between SCT-determinants and proximal goal to do the tick check (DV)

	*β*	*t*	*R* ^*2*^	*R* ^*2*^ change
DV: Proximal goal to do the tick check				
Model 1:			.02	.01
Self-efficacy	.12	2.61^**^		
Model 2:			.25	.23
Self-efficacy	.02	.41		
Outcome expectations:				
Perceived severity	.01	.16		
Response efficacy	.04	0.78		
Self-evaluative	.43	8.05^**^		
Injunctive norm	.04	.93		
Descriptive norm	.02	.46		
Model 3:			.25	.00
Self-efficacy	.02	.50		
Outcome expectations:				
Perceived severity	.01	.13		
Response efficacy	.04	.83		
Self-evaluative	.43	7.97^**^		
Injunctive norm	.04	.91		
Descriptive norm	.02	.46		
Knowledge	−.01	−.16		
Experience	−.02	−.47		

All models controlled for gender (*R* ^*2*^ = 0.01); ^**^ *p* < 0.01, ^*^ *p* < 0.05

## Discussion

The aim of the present study was to examine the determinants of the single most effective behavior to prevent Lyme disease, which is performing the tick check [8, 10]. The survey questionnaire was based on existing evidence from the field of Lyme preventive behaviors, and on SCT [16, 17], which has a firm evidence base in predicting and changing health behaviors [17]. This study was warranted because a review of existing literature revealed, first, little evidence of the effectiveness of existing interventions; second, mostly studies without a clear definition of the term ‘preventive behaviors to avoid Lyme disease’, or did not concretize which systematic steps should be followed in order to perform a tick check effectively; third, the use of behavioral theory is largely lacking, even though using theory is of great scientific and practical benefit.

Proximal goal, self-efficacy, and social as well as self-evaluative outcome expectations contributed significantly to performing the tick check. Experience with ticks and/or Lyme disease added to the explained variance in doing the tick check, above and beyond the SCT variables, albeit to a small extent. The full model explained 28% of the variance in doing the tick check. This is in line with literature showing that, in general, social-cognitive models of behavior explain approximately 30% of the variance in behavior [32]. A more recent example is a meta-analysis showing that SCT explains on average 25% of the variance in physical activity [33].

As SCT posits that the proximal goal to perform a behavior partially explains the effects of the other determinants [16, 17], and because it was the strongest predictor of the tick check, we also examined multivariate associations between proximal goal (DV) and self-efficacy, outcome expectations, knowledge and experience (IV’s). Results revealed that only self-evaluative outcome expectations were associated with the proximal goal to do the tick check, confirming its central place in the model. By itself, self-evaluative outcome expectations explained 23% of the variance in the proximal goal. Furthermore, this indicates that self-efficacy, social outcome expectations and experience only have direct associations with doing the tick check, thus not through associations with the proximal goal to do so.

### Implications for theory and practice

Because our findings are based on SCT and existing evidence, we are able to formulate a number of theoretical and practical implications:

Proximal goal is the strongest predictor of tick check performance, which is in line with SCT [16, 17], as well as with many other studies showing that setting goals (e.g. [34]) or forming intentions (e.g. [35]) is strongly related to subsequent behavior. However, to date, goal-setting seems to have been largely ignored in this field of behaviors to prevent Lyme disease. Future interventions should therefore stimulate explicit goal-setting. In that respect, implementation intentions have shown to be especially effective, because they specify the critical situation in which the behavior needs to be performed, such that the situation becomes a cue for behavior [36]. People should thus be induced to state when and where they will perform the tick check, e.g. “When I come home from my visit to the forest, I will do the tick check in our bathroom.”

The strong direct effect of self-efficacy on tick check performance is in line with SCT, as well as with previous studies on preventive behaviors [20, 21]. Theoretically, it is rather surprising that self-efficacy has no association with proximal goal. The exclusive direct association with behavior may indicate that actual and perceived control over the behavior play an important role in performance, perhaps because of (actual or perceived) impediments, such as forgetting or not having privacy or tools, notably a mirror and tweezers. Translating this finding into a practical recommendation, modeling is a potential effective behavior change method. That is because a central feature of SCT is its focus on the human capacity for observational learning, which means that people learn behaviors by watching others perform that behavior [37]. Observational learning has multiple effects, one being acquiring skills and gaining confidence in one’s ability to perform a particular behavior (i.e. self-efficacy). Especially showing a ‘coping’ model, who successfully struggles with and then overcomes impediments, increases self-efficacy of people who are learning complex new behaviors [37]. McAlister et al. [37] provide a comprehensive list of additional behavior change methods to improve skills and self-efficacy, such as guided practice, verbal persuasion and planning coping responses, that are potentially effective for application in interventions to promote the tick check.

Self-evaluative outcome expectations was the only determinant explaining the proximal goal to do the tick check, as well as the strongest outcome expectation related to actual performance. This indicates that people perform the tick check to feel good about themselves, and less because of explicit health concerns (also see: physical outcome expectations). Practically, this means that interventions are potentially effective when they show that performing the tick check results in positive feelings about the self. This could be done with messages that convey this idea directly (e.g. “Do the tick check and feel good about yourself!”). However, it is likely more effective to show how other people feel good about themselves after performing the tick check, because through observational learning, people also learn about the potential rewards of a behavior. Thus again, modeling the tick check can be an effective method, by showing that the models feel good about themselves after performing the tick check.

Social outcome expectations were the fourth and final determinant associated with performing the tick check. However, whereas the descriptive norm had a positive relationship with the tick check, the injunctive norm showed a negative relationship. This can be explained by findings that descriptive and injunctive norms represent different types of motivation for behavior [38]. Descriptive norms motivate behavior by providing information about what *is* the best course of action in a given situation. Underlying is the heuristic that it must be sensible or instrumental what most other people are doing, otherwise they would not be doing it. In contrast, injunctive norms specify what *ought* to be done in order to gain social approval (or prevent social disapproval), and thus to be socially accepted [38]. Thus, the results of our study show that the perception that others are doing the tick check is related to performing it oneself, presumably because this conveys the idea that this is a ‘good’ course of action. However, the belief that others approve of doing the tick check is not related to actual performance, indicating that the tick check is not performed (or even less so) when people expect to gain social acceptance through that behavior. Recent studies have confirmed the differential effects of descriptive and injunctive social norms, e.g. [39]. For intervention development, these results indicate that again modeling can be an effective behavior change method, because this is in essence showing that others do the tick check. Moreover, it is important not to convey in any way that other people will approve of doing the tick check (or disapprove of not doing the tick check).

Surprisingly, none of the physical outcome expectations were associated with the tick check. We were thus not able to confirm the impact of perceived susceptibility and severity on performance of the tick check, even though the literature indicates that both are determinants of preventive behaviors, e.g. [12, 13, 20]. Unfortunately, we were unable to reliably measure perceived susceptibility, which was therefore omitted from the analyses. Perceived severity was included, but did not have any multivariate associations with performing the tick check or the proximal goal to do so. This may indicate that measurement of severity was also suboptimal, even with an acceptable Cronbach’s alpha. However, it may also mean that effects of these risk perceptions are indeed weak. The literature about risk perceptions is somewhat mixed. For example, Brewer et al. [40] conclude, based on their meta-analysis that susceptibility and severity are rightfully core concepts in theories of health behavior. However, the target behavior in their meta-analysis is vaccination, a discrete and single behavior and relatively easy to perform [40], which are not characteristics of performing the tick check. Moreover, in another meta-analysis it is concluded that threatening or fear-arousing messages (i.e. fear appeals) are effective in changing behavior *only* under high self-efficacy [41].

This has important implications for intervention development, as our study shows that self-efficacy is indeed a key determinant of doing the tick check. Because self-efficacy shows high variability, it is recommended not to use threatening information in mass media campaigns. Addressing the severity of and susceptibility to Lyme disease in interventions should thus be used cautiously.

In light of the lack of associations between risk perceptions and the tick check it may not be surprising that response efficacy showed no association either, as this reflects perceptions about the efficacy of the tick check to prevent those risks. This also goes for the non-significant results of knowledge, that reflected instrumental knowledge of ticks and Lyme disease. Practically, and based on our results, interventions that aim to increase doing the tick check can certainly include education, but the core should be a positive campaign, showing how to do the tick check, showing that others do the tick check as well as showing that doing the tick check results in feeling good about oneself. This could be supplemented with strategies for setting the goal to do the tick check.

In sum, the associations between the tick check and proximal goal, self-efficacy, self-evaluative outcome expectations, and descriptive norm are in line with theory and provide clear implications for future interventions. However, a number of findings were not in line with our theoretical model, and the implications thereof warrant further discussion.

### Strengths and limitations

The strengths of the present study include the large sample size and that it was theory-based. In addition, our study is among the first to clearly describe the tick check step-by-step, which is important for health promotion and research purposes. Although a convenience sample was used, most participants were recruited through a website for people who are interested in nature. Therefore, our sample may not be representative for the whole population, but instead for people who regularly visit nature and make use of the internet and are thus likely to come into contact with ticks, thus representing the target group of interest.

Limitations that should be noted are, first, the cross-sectional nature of our study, which does not allow for statements regarding causality. Because of this, the implications for practice should be interpreted with caution. Furthermore, because data gathering in this study was done by means of a survey questionnaire, the data in this study was self-reported. This may have introduced memory bias and/or social desirability bias. Finally, this study was conducted in late autumn and winter. Ticks are often associated with warm weather and summer. This may have influenced participants’ answers on statements about their current tick check behavior and their proximal goal to do the tick check in the coming month.

### Future research

Based on our literature study, an overall recommendation is to base future studies on established behavioral theory. Following guidelines for intervention development, e.g. [14], the first step is to clearly define the behavior of interest of the target group. Furthermore, this entails multifactorial testing determinants of that behavior (in this case doing the tick check), because only then the relative strength of determinants can be established. In contrast, testing only one class of determinants (e.g. knowledge, or risk perceptions) may result in overestimating associations with doing the tick check.

Derived from findings of the current study, we recommend experimental testing of our practical recommendations. This would allow for verifying the current results (i.e. whether identified determinants are indeed causes of doing the tick check), as well as directly inform intervention development. Specifically, future research could test the effects of modeling on self-efficacy, self-evaluative outcome expectations and descriptive social norm on tick check performance, as well as on actual performance of the tick check. Another promising avenue is testing whether goal-setting strategies are an effective intervention tool in this context. As an example, Beaujean and colleagues have recently tested whether a theory-based intervention using a leaflet or a movie are effective in strengthening determinants of preventive behavior against Lyme disease [42]. Results showed that both the leaflet and movie were valued by participants, and led to post-intervention increases in the intention to do the tick check, but not at follow-up.

## Conclusion

Our study identifies behavioral determinants that are associated with tick check performance, as well as determinants that are not – or even negatively – associated. Survey questionnaire results revealed that proximal goal, self-efficacy, and social as well as self-evaluative outcome expectations contributed significantly to performing the tick check. Beyond the SCT variables, experience with ticks and/or Lyme disease added to the explained variance in doing the tick check as well, although this addition was small. The full model explained 28% of the variance in doing the tick check. These results have implications for health communication activities that attempt to promote performing the tick check among the general public.

## Additional file

Additional file 1: Survey questionnaire. (DOCX 21 kb)

## Abbreviations

DV: dependent variable
IV: independent variable
M: mean
SCT: social cognitive theory
SD: standard deviation

## Footnotes

1 The databases Web of Science, Scopus and Google Scholar were searched. The search terms that were used are “preventive measures”, “protective measures”, “preventive behavior”, “protective behavior”, “tick bites”, “ticks”, “tick check”, “Lyme disease” and “determinants”. The search period included all years. The date of search was September, October and November 2015. Articles were selected as relevant for this literature study if a topic of the study was about the determinants of behavior of taking preventive measures or specifically doing a tick check to avoid tick bites and Lyme disease. In total, eleven articles were included.
