# Supplementary material for: Social-cognitive determinants of the tick check: a cross-sectional study on self-protective behavior in combatting Lyme disease
Source: BMC Public Health. 2017 Nov 25;17:900. doi: 10.1186/s12889-017-4908-1 (PMC5702127; doi:10.1186/s12889-017-4908-1)
Supplement: Additional file 1: — Survey questionnaire. (DOCX 21 kb) [file 12889_2017_4908_MOESM1_ESM.docx]

**Determinants of the tick check**

Q1 Dear respondent,

Thank you for your interest in this survey! This survey is about doing a tick check after visiting nature. The survey is conducted by a student of Wageningen University as part of a master thesis under supervision of dr. B. C. Mulder of the chair group Strategic Communication, and dr. A. J. H. van Vliet of the chair group Environmental System Analysis and Tekenradar.nl. The results of the survey are anonymous and will not be provided to third parties. Taking part in the survey will take about fifteen minutes. On the next page, information about the tick check will be provided.

Thank you for your participation!

Q2 Performing the tick check means that you check your body for ticks after visiting nature, like forest, moorland, park or garden. Ticks are small, spider-like animals that live in nature in for example high grass and bushes. If they bite, ticks can cause Lyme disease. Therefore, it is important to check your body for ticks after visiting nature and to remove them from your skin as quickly as possible. We call this the tick check. The tick check consists of the following steps. Please read them carefully.

1. Remove all your clothes in order to inspect your entire skin well. Ticks usually go to warm places, such as the armpits, knees, the buttocks and behind your ears.
2. Check systematically, from top to bottom or bottom to top, whether there are ticks on your body. You can use a mirror for places that are difficult to see.
3. If a tick is found, remove it with pointy tweezers or a tick remover. If you use pointy tweezers, grab the tick firmly by its head and pull it out straight. If you use a tick remover, follow the instructions that come with the remover.

Q3 This question is about your current behavior regarding the tick check. Please indicate the extent to which the following statements apply to you. Each statement starts with “During the past month, when I had visited nature...”.

1 = Never
100 = Always

During the past month, when I had visited nature...

______ ...I checked my body for ticks. (1)

______ ...I systematically checked, from top to bottom or bottom to top, my body for ticks. (2)

______ ...I used a mirror to look at places of my body that are difficult to see, while doing the tick check. (3)

______ ...I used a tick remover or pointy tweezers to remove the tick, when I found a tick on my skin. (4)

Q4 This question is about how confident you are that you are able to perform certain actions. Please indicate how confident you are that you can perform the actions in each statement on a scale of 1 to 100.

1 = Definitely not
100 = Definitely yes

______ I can undress completely to look at my entire skin well. (1)

______ I am able to systematically check my body for ticks, from top to bottom or bottom to top, if necessary with the use of a mirror. (2)

______ I am able to recognize a tick and find it on my skin. (3)

______ If I find a tick, I can remove it from my skin correctly. (4)

______ I am able to completely perform all the steps of the tick check, as described earlier in this survey. (5)

______ I can perform the tick check completely when I am tired. (6)

______ I can perform the tick check completely if I have little time. (7)

______ I am able to perform the tick check completely when I have other things on my mind. (8)

______ I am able to perform the tick check when I am in company of other people. (9)

______ I can perform the tick check completely when I am on vacation. (10)

______ I can perform the tick check completely in hot weather. (11)

______ I can perform the tick check completely in cold weather. (12)

______ I can perform the tick check completely if I do not have a mirror with me. (13)

______ I am able to perform the tick check completely if I do not have pointy tweezers or a tick remover with me. (14)

______ I am able to remember that I have to do a tick check after I visited nature. (15)

Q5 This question is about your knowledge and experience with regard to ticks, the tick check and Lyme disease. If you think a statement is true, fill in ‘True’; if you think a statement is not true, fill in ‘Not true’. If you do not know the answer, fill in ‘I don’t know’.

|  | True (1) | Not true (2) | I don’t know (3) |
| --- | --- | --- | --- |
| The bacteria that causes Lyme disease, can be transmitted through an infected tick. (1) |  |  |  |
| Lyme disease can lead to nerve disorders, joint problems and/or heart problems. (2) |  |  |  |
| Ticks are at least 1 centimeter tall. (3) |  |  |  |
| If a tick is removed from the skin within 24 hours, the chances of getting Lyme disease are reduced. (4) |  |  |  |
| Ticks can be active at temperatures below 15 °C. (5) |  |  |  |
| Ticks can be present in the forest. (6) |  |  |  |
| Ticks can be present in gardens. (7) |  |  |  |
| Sometimes, ticks are only 1 millimeter tall. (8) |  |  |  |
| I have been bitten by a tick before. (9) |  |  |  |
| I have Lyme disease or have had it before. (10) |  |  |  |
| I know someone who has Lyme disease or has had it before. (11) |  |  |  |
| Before I started this survey, I already knew about the existence of the tick check. (12) |  |  |  |
| Before I started this survey, I already knew how to properly do the tick check. (13) |  |  |  |

Q6 This question is about your attitude regarding ticks, the tick check and Lyme disease. Please indicate the extent to which you agree with the statements.

1 = Totally disagree
100 = Totally agree 

______ I am afraid of ticks. (1)

______ I am disgusted by ticks. (2)

______ If I had Lyme disease, I would be worried. (3)

______ Lyme disease would have a negative impact on my life. (4)

______ I believe Lyme disease is difficult to cure. (5)

______ I think Lyme disease is a serious condition. (6)

______ There is a high risk that I will be bitten by a tick, if I visit nature. (7)

______ I think there is a great chance that I will contract Lyme disease after a tick bite. (8)

______ I think doing a tick check is important for my health. (9)

______ My risk of being bitten by a tick is equal to the risk of other people. (10)

______ Doing a tick check prevents me from getting Lyme disease. (11)

______ A tick check is an effective way to prevent getting Lyme disease. (12)

______ Compared to others, I think I have a higher risk of being bitten by a tick. (13)

______ Doing a tick check brings me many benefits, such as reducing the chance of Lyme disease and possibly even removing a tick before it bites. (14)

______ Doing a tick check is bothersome, for example because it takes a lot of time or because it is unpleasant to do. (15)

______ The benefits of performing a tick check are more important to me than the inconvenience that it may cause. (16)

______ Compared to others, I think I have a smaller risk of being bitten by a tick. (17)

______ People whose opinion I value, will appreciate it if I do the tick check. (18)

______ People whose opinion I value, will disapprove if I do the tick check. (19)

______ People whose opinion I value, perform the tick check after having visited nature. (20)

______ Performing a tick check is important to me. (21)

______ By doing a tick check I feel good about myself. (22)

Q7 People can have different reasons for performing the tick check. Could you indicate how important the following reasons for performing the tick check are for you?

1 = Not important at all
100 = Very important

______ My degree of disgust or fear of ticks. (1)

______ The severity of Lyme disease. (2)

______ The extent to which I am at risk of being bitten by a tick when I visit nature. (3)

______ The benefits a tick check can yield, like not getting Lyme disease and possibly even removing a tick before it bites. (4)

______ The disadvantages a tick check can yield, like having to undress completely and needing to have a pair of tweezers. (5)

______ What people whose opinions are important to me think when I do the tick check. (6)

______ How good I feel about myself after doing a tick check. (7)

Q8 What are reasons for you to (possibly) have a positive attitude towards the tick check?

Q9 What are reasons for you to (possibly) have a negative attitude towards the tick check?

Q10 What would withhold you from doing the tick check?

Q11 These statements are about what you would do if you would visit nature right now or in the coming month. Each statement starts with “In the coming month, if I visited nature...” Please indicate the extent to which you agree with the statements.

1 = Totally disagree
100 = Totally agree

In the coming month, if I visited nature...

______ ... I will try to do the tick check. (1)

______ ... I really plan to do the tick check. (2)

______ ... I will definitely do the tick check. (3)

______ ... I will try to use pointy tweezers or a tick remover if I discover a tick bite. (4)

______ ... I really plan to use pointy tweezers or a tick remover if I discover a tick bite. (5)

______ ... I will definitely use pointy tweezers or a tick remover if I discover a tick bite. (6)

______ ... I will try to use a mirror when doing the tick check. (7)

______ ... I really plan to use a mirror when doing the tick check. (8)

______ ... I will definitely use a mirror when doing the tick check. (9)

Q12 Please give a short explanation of your answer to the previous question: why do you think you will or will not do the tick check?

Q13 Do you live in the Netherlands?

- Yes (1)
- No (2)

Q14 What is your age?

Q15 What is your gender?

- Man (1)
- Woman (2)

Q16 What is your highest level of education?

- Primary school (1)
- High school (2)
- Intermediate vocational school (3)
- Higher vocational education / University (4)

Q17 On average, how often do you visit nature? (Like forest, moorland, park or garden)

- Daily or weekly (1)
- Monthly (2)
- A few times per year (3)
- Yearly or less than once per year (4)

Q18 Where did you find this survey?

- Nature Today (1)
- Facebook (2)
- Tekenradar (3)
- Other (4)
